# Evaluating Age-Friendly Intergenerational Classrooms: Insights From Instructors and Students

**DOI:** 10.1093/geroni/igaa057.1766

**Published:** 2020-12-16

**Authors:** Joann Montepare

**Affiliations:** Lasell University, Newton, Massachusetts, United States

## Abstract

Age-friendly University (AFU) campuses are reshaping how we think about teaching and learning in higher education. In particular, intergenerational classrooms are on the rise as shifting age demographics call for institutions to create new opportunities for older learners and encourage intergenerational exchange. Age diverse classrooms have distinctive needs and dynamics that instructors, and students, will need to learn how to navigate. This presentation will describe outcomes of one AFU institution’s attempt to identify the challenges and triumphs of intergenerational classrooms through facilitated instructor and student reflections in different classrooms over the course of several semesters. Recommendations will be offered for enhancing intergenerational exchange in classrooms across disciplines, as well as evaluating attitudes, logistics, and learning outcomes. Part of a symposium sponsored by Intergenerational Learning, Research, and Community Engagement Interest Group.

